# Variables Influencing Differences in Sequence Conservation in the Fission Yeast *Schizosaccharomyces pombe*

**DOI:** 10.1007/s00239-021-10028-y

**Published:** 2021-08-26

**Authors:** Simon Emanuel Harnqvist, Cooper Alastair Grace, Daniel Charlton Jeffares

**Affiliations:** Department of Biology, University of York, York, YO10 5DD UK

## Abstract

Which variables determine the constraints on gene sequence evolution is one of the most central questions in molecular evolution. In the fission yeast *Schizosaccharomyces pombe*, an important model organism, the variables influencing the rate of sequence evolution have yet to be determined. Previous studies in other single celled organisms have generally found gene expression levels to be most significant, with numerous other variables such as gene length and functional importance identified as having a smaller impact. Using publicly available data, we used partial least squares regression, principal components regression, and partial correlations to determine the variables most strongly associated with sequence evolution constraints. We identify centrality in the protein–protein interactions network, amino acid composition, and cellular location as the most important determinants of sequence conservation. However, each factor only explains a small amount of variance, and there are numerous variables having a significant or heterogeneous influence. Our models explain more than half of the variance in dN, raising the possibility that future refined models could quantify the role of stochastics in evolutionary rate variation.

## Introduction

The question of which variables determine the rate of sequence evolution is one of the most central in evolutionary genomics. While there is a long list of variables that are believed to influence the rate of sequence evolution, the importance of each has still not been explored in fission yeasts as far as we are aware. This has however been examined in a range of other organisms, generally showing that gene expression levels most strongly influence sequence constraint at least in single-celled organisms (Zhang and Yang 2015). Because of the significance of *Schizosaccharomyces pombe* in genetic research, it is important to investigate whether the drivers of molecular evolution differ in fission yeasts compared to other organisms.

Determining how much of the variance in sequence evolution rates is determined by each variable is a challenging high-dimensional regression problem. Previous studies (Jovelin and Phillips 2009; Yang and Gaut 2011; Alvarez-Ponce et al. 2017) have used principal components regression (PCR) to determine the influence of each variable. We propose the additional use of the similar partial least squares regression (PLS), which unlike PCR reduces dimensionality of dependent variables with respect to both the independent and dependent variables rather than just the dependent variables (Haenlein and Kaplan 2004). Both methods are highly interpretable; in PCR, the variance in the independent variable explained by each dependent variable can be calculated (Drummond et al. 2006), whereas in PLS, the variable importance in projection (VIP) (Mehmood et al. 2012) provides an indicator of variable importance. Additionally, partial correlation analysis, which finds the correlation between two variables adjusting for the influence of covariates, is used as a second estimate of the influence of each variable.

We use publicly available data from Grech and colleagues (Grech et al. 2019), with additional data from the PomBase (Lock et al. 2019), AnGeLi (Bitton et al. 2015), and STRING (Szklarczyk et al. 2019) databases to model the influence of 170 genomic, proteomic, and functional variables on gene conservation. Using the modelling techniques described, we attempt to determine which variables are most influential on gene conservation in fission yeast.

## Background: Variables Influencing Sequence Constraints

### Functional Importance

Kimura and Ohta (1974) suggested, based on the neutral theory of molecular evolution (Kimura 1968) that functional importance (importance of gene for organismal fitness) would be the most important predictor of sequence evolution constraint. This hypothesis is intuitive; deleterious mutations in highly important genes should be more detrimental to fitness, providing a selective pressure against sequence change. However, once confounders are adjusted for, it seems that functional importance measured as knockout fitness or survival only has a minor impact on sequence evolution rates (Zhang and Yang 2015). There are good reasons for this rather counterintuitive finding. It has been suggested (Bergmiller et al. 2012) that the reason ‘essential’ genes can be lost in evolution is that other genes replace their function. Aguilar-Rodríguez and Wagner (2018) found that, in the context of metabolic networks, the rate of evolution is strongly correlated with ‘superessentiality’, i.e. how easily the protein can be bypassed in the network. Additionally, Pal et al. (2006) argue that essentiality under laboratory conditions may be entirely different from essentiality under natural (less favourable) conditions, although in the case of fission yeast Grech et al. (2019) found that constraint estimated from saturating transposon mutagenesis in the laboratory correlated well with evolutionary constraint. Perhaps more significantly, dispensability and essentiality both refer to the effect of gene loss rather than point mutations, which is how sequence change is measured (Pal et al. 2006; Alvarez-Ponce 2014).

### Gene Expression

In the past two decades, gene expression has emerged as the strongest predictor of sequence conservation, at least in unicellular organisms (Alvarez-Ponce 2014). The so-called expression–evolutionary rate (E-R) anticorrelation has been reported across a range of organisms (Alvarez-Ponce 2014; Zhang and Yang 2015), and conserved genes have indeed been found to be highly expressed in *Schizosaccharomyces pombe* (Mata 2003). There are multiple suggestions why this might be, and Alvarez-Ponce (2014) and Zhang and Yang (2015) explain these in detail. They include hypotheses that the high codon bias of highly expressed genes introduces high constraints (Akashi 2001, 2003), that reduction in organismal fitness resulting from deleterious mutations is correlated with expression level (Rocha and Danchin 2004), that the cost of producing non-functional proteins increases with protein abundance (Cherry 2010; Gout et al. 2010), that highly expressed proteins prefer residues that reduce the risk of misinteraction (Yang et al. 2012), and that the mRNAs of highly expressed genes are more strongly folded which increases constraint (Park et al. 2013). However, the hypothesis that probably has most evidential support (Alvarez-Ponce 2014) is the translational robustness hypothesis (Drummond et al. 2005). It suggests that some proteins may have the ability to fold correctly even if the wrong amino acids have been added as a result of translational errors, and that these proteins are more constrained in order to preserve this translational robustness. Because the negative consequences of mistranslation (e.g. misinteraction and misfolding) would be more severe in highly expressed proteins, this selective pressure would be expected to increase with expression levels. Notably, these hypotheses are not mutually exclusive, and it seems likely that more than one of them contribute to this widely noted relationship.

### Network Centrality

One hypothesis that has been found to be very important in some studies, but not in others, is that proteins that are more central in the protein–protein interactions network are more conserved. This, as suggested by Ingram (1961), is generally thought to be because mutations in highly connected proteins that result in loss of binding to other proteins would cause greater disruption to a greater number of pathways than less central proteins. Indeed, network centrality has been found to be one of the most important determinants of the rate of sequence evolution in organisms as diverse as humans (Alvarez-Ponce et al. 2017) and *Saccharomyces cerevisiae* (Fraser et al. 2002). Mannakee and Gutenkunst (2016) used systems modelling to develop a metric, *dynamical influence,* which measures functional importance of the protein within its interactions network. They found that this metric showed one of the strongest correlations with evolutionary rate, comparable with expression levels, even once covariates had been adjusted for. Overall, there is strong and increasing evidence that network centrality may be as important as expression levels in determining the rate of evolution; it may be that in some cases, protein–protein interaction network centrality is more important. However, when considering other networks or specific sub-networks, it is far less straightforward. For instance, central transcription factors generally evolve more quickly than peripheral ones (Jovelin and Phillips 2009). Aguilar-Rodríguez and Wagner (2018) found that in bacterial metabolic networks, enzymes’ rates of evolution depend more on their function in the network than their centrality.

### Sequence Length

The correlation between sequence length and conservation appears to depend greatly on context; some studies find no correlation and for those that do, there is no agreement on the direction of correlation (Alvarez-Ponce 2014). Alvarez-Ponce (2014) points out that the Hill-Robertson effect (Hill and Robertson 1966), which is that linkage between closely located genes reduces the efficiency of selection, is stronger for longer genes. Longer genes do however generally contain more introns, which reduces the Hill-Robertson effect (Comeron and Kreitman 2000).

### Other Variables

Finally, a host of other variables influencing the rate of sequence evolution in a range of organisms have been reported before, including chaperone dependence (Rutherford 2003) which has been found to be the most important determinant in *Saccharomyces cerevisiae* (Alvarez-Ponce et al. 2019), pleiotropy (Hahn and Kern 2005), cellular location (Julenius and Pedersen 2006; Liao et al. 2010), and codon bias (Drummond and Wilke 2008). Zhang and Yang (2015) and Alvarez-Ponce (2014) provide comprehensive reviews of the various variables implicated in sequence evolution.

## Methods

### Sequence Alignments

Proteomes of the four fission yeasts, *Schizosaccharomyces pombe*, *S. japonicus*, *S. octosporus*, and *S. cryophilus* were downloaded from NCBI and assigned to orthogroups by Orthofinder 2.3.11 (Emms and Kelly 2019) with default parameters. Corresponding transcriptomes were also downloaded from NCBI and sorted into orthogroups according to Orthofinder results. Orthogroup nucleotide sequences were aligned into codons using corresponding protein alignments with MACSE 2.05 (Ranwez et al. 2011) and default parameters. Unreliable sites were filtered out with Gblocks (Castresana 2000).

### Estimation of ω and d*N*

**Fig. 1:**
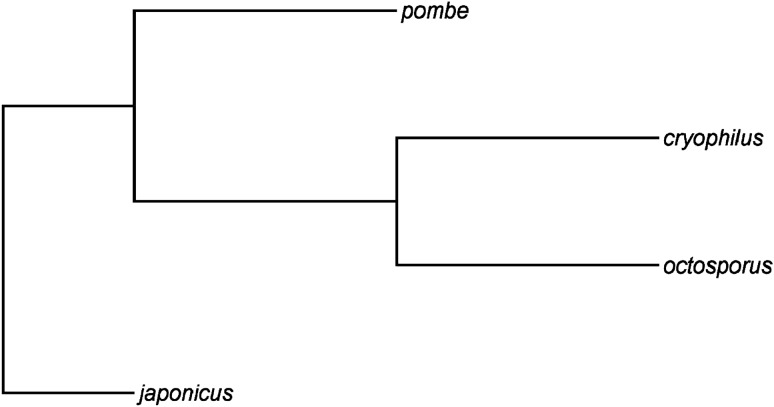
Maximum likelihood phylogenetic tree of the four fission yeast (*Schizosaccharomyces*) species based on a concatenated alignment of 50 orthologous groups, chosen at random. Created with MEGA 10.2.5 using the Tamura-Nei model assuming uniform rates across all sites, using Nearest-Neighbor-Interchange as ML heuristic and rooted with the *S. japonicus* sequences. Visualised with FigTree 1.4.4 (tree.bio.ed.ac.uk/software/figtree/)

We attempted to calculate the ratio of nonsynonymous substitutions to synonymous substitutions (d*N*/d*S*, or ω), which is the most common method of estimating the rate of molecular evolution. This was performed using CODEML (Yang 2007) with an M0 model (NSsites = 0, model = 0) using the described alignments. Only 1:1:1:1 orthologous groups were used, with one gene per species. For all analysis in CODEML, we used a nearest-neighbour interchange maximum likelihood phylogenetic tree (Fig. 1) created in MEGA (Tamura et al. 2021) with a Tamura-Nei model assuming uniform rates across all sites. This tree was generated from an alignment of 50 concatenated orthologs chosen at random. Unfortunately, d*S* was estimated to have a mean of 12 indicating saturation, meaning that ω could not be reliably estimated. This is because the genetic distance between the four fission yeasts is too great, as previously reported by Fawcett et al. (2014). We chose to retain the calculated d*N* values (*n* = 2576) as a metric of evolutionary rate, as there was no indication that this rate was saturated. It must however be remembered that since this rate does not adjust for the rate of synonymous substitutions, results should be treated with caution.

### phyloP

Additionally, we relied on “phylogenetic *p*-values”, phyloP (Siepel et al. 2006), a hidden Markov model approach to detect selection and its direction. It models sequence differences between species, per nucleotide site, under a null hypothesis of neutral evolution. In other words, it indicates the probability of seeing the observed sequence changes given no selection. The absolute values of phyloP are − log_10_(*p*-values), and negative scores indicate acceleration (positive selection) whereas positive scores indicate conservation (negative selection). Therefore, extreme phyloP values indicate strong evidence for selection. We used values (*n* = 5181) already available from Grech et al. (2019), who calculated the average phyloP per gene, using only coding sequences, for *S. pombe* with the HAL toolkit (Hickey et al. 2013) using DNA alignments of *S. pombe*, *S. cryophilus*, *S. octosporus*, and *S. japonicus.*

### Data Sources

A list of genes (*n* = 5181) of protein coding genes in the *Schizosaccharomyces pombe* genome, with data on constraint (calculated as above), gene and protein expression, gene length, chromosome, essentiality and fitness on solidum medium, were retrieved from the Figshare repository of Grech et al. (2019). Interactome data, both for direct physical interactions as well as for functional (i.e., all) interactions were retrieved from STRING (Szklarczyk et al. 2019). Further data on intron number, average intron length, and genomic location were retrieved from AnGeLi (Bitton et al. 2015). Gene ontology data (‘GO slims’, which are groups of broad categories of GO Terms) were retrieved using the GO Term Mapper (go.princeton.edu/cgi-bin/GOTermMapper), with the gene list from Grech et al. as input. All other data, including amino acid composition and protein length and size, were retrieved from PomBase (Lock et al. 2019). The links to the datasets are available in the “Availability of data and material” section.

### Data Pre-processing

Gene ontology (GO) annotation slims as well as chromosome data were one-hot encoded such that each GO slim became a column with the values in each row being either 0 (corresponding to “false”) or 1 (“true”). This was done using the mltools 0.3.5 R package (Gorman 2018). All other variables were continuous. Amino acid composition data were scaled to proportion of protein. Missing data were imputed using missForest 1.4 (Stekhoven and Buhlmann 2012) using all columns except constraint (dependent variable), GO slims, and amino acid composition, as the two latter required too much compute power. All preprocessing was carried out in R 4.1.0 (R Core Team 2020) with use of Tidyverse 1.3.1 (Wickham et al. 2019). Some variables were removed due to low variance (i.e. if they contained missing values after scaling).

### Network Analysis

Two protein–protein interactions network graphs were constructed from STRING (Szklarczyk et al. 2019), a database containing both experimentally confirmed and predicted interactions. The first network included all interactions, whether direct or indirect, and the second only direct, physical interactions. The graphs were created in igraph 1.2.6 R package (Csardi and Nepusz 2006), using a minimum STRING interaction score of 0.400 to filter out unreliable interactions. The centrality metrics used were betweenness, closeness, degree (all proposed by Freeman 1979) and eigenvector centrality (Bonacich 1972). Closeness centrality is the inverse of the average shortest distance to all other nodes, and betweenness centrality is the number of closest paths between all pairs of nodes on which the node is located; these two global metrics provide a measure of control flow through the network (Alvarez-Ponce et al. 2019). Degree centrality (also known as ‘connectivity’) is simply the number of direct neighbours a node has, and is a local metric of centrality (Alvarez-Ponce et al. 2019). Eigenvector centrality captures both local and global information and is a weighted sum of the centralities of all the nodes that node is connected to (Negre et al. 2018).

### Regression Models

To model sequence evolution constraint (phyloP) by all independent variables, we fitted partial least squares (PLS) and principal components regression (PCR) models. Both PLS and PCR models were constructed using the pls R package v 2.7-3 (Mevik et al. 2019) and optimum number of components selected automatically with the selectNcomp function. All data were centred and scaled. Ten-fold cross-validation was used in training to guard against overfitting. Performance prediction was then made on a held out test set; the size of this was set to 30% of the dN dataset (*n* = 2576), and the same genes were used for the phyloP test set, corresponding to just under 15% of that dataset (*n* = 5181).

### Variable Importance Estimation

Two very similar methods were used to interpret the PCR and PLS models. For the PCR model, the *percent variance explained by each variable* was calculated by summing each variable’s influence on the latent projections in each model component, scaled by the variance in the dependent variable explained by that component (Drummond et al. 2006). For PLS, *variable importance in projection* (VIP) scores were calculated using plsVarSel 0.9.6 (Mehmood et al. 2012). When VIP is used for variable selection, it is generally agreed that variables with VIP values below 1 can be removed (Chong and Jun 2005) which provides a guide for interpretation. The difference between the two scores is that while the variance explained by PCR only estimates how well the model describes the dependent variable, PLS-VIP estimates how well the model describes both independent and dependent variables (Andersen and Bro 2010), which is useful when making inferences about variable influence.

### Model Comparisons

As comparison against the other regression models, a random forest (RF) model was trained using randomForest 4.6-14 (Liaw and Wiener 2002). It was trained using the default hyperparameters of 500 trees and the number of variables available for splitting at each node (“mtry”) set to the number of variables divided by 3. Root mean squared error on the 20% holdout test set was calculated for each model using the rmse function in the Metrics package (Hamner and Frasco 2018). Variance explained by PLS/PCR models was calculated with the R2 function in pls. Variance explained by the random forest model was calculated using ‘pseudo *R*^2^’ (Liaw and Wiener 2002):

$${\text{variance explained}} = 1 - \frac{{{\text{MSE}}\left( {Y, \hat{Y}} \right)}}{{{\text{variance}}\left( Y \right)}}$$

where MSE is the mean squared error of prediction on training data, *Y* is the independent variable vector (true values), and $\hat{Y}$ the vector of predicted values.

### Partial Correlation Analysis

Partial correlations were calculated between each independent variable and constraint (d*N* or phyloP score), using variables in all other variable groups (but not variables in the same variable group) as covariates. Spearman’s rank correlation coefficient was chosen as a nonparametric alternative to Pearson’s correlation coefficient. Calculations were performed using the Pingouin 0.3.12 package (Vallat 2018) in Python 3.8.5. Bonferroni correction was used to adjust for multiple comparisons.

## Results and Discussion

### Network Centrality Increases Constraint

**Fig. 2:**
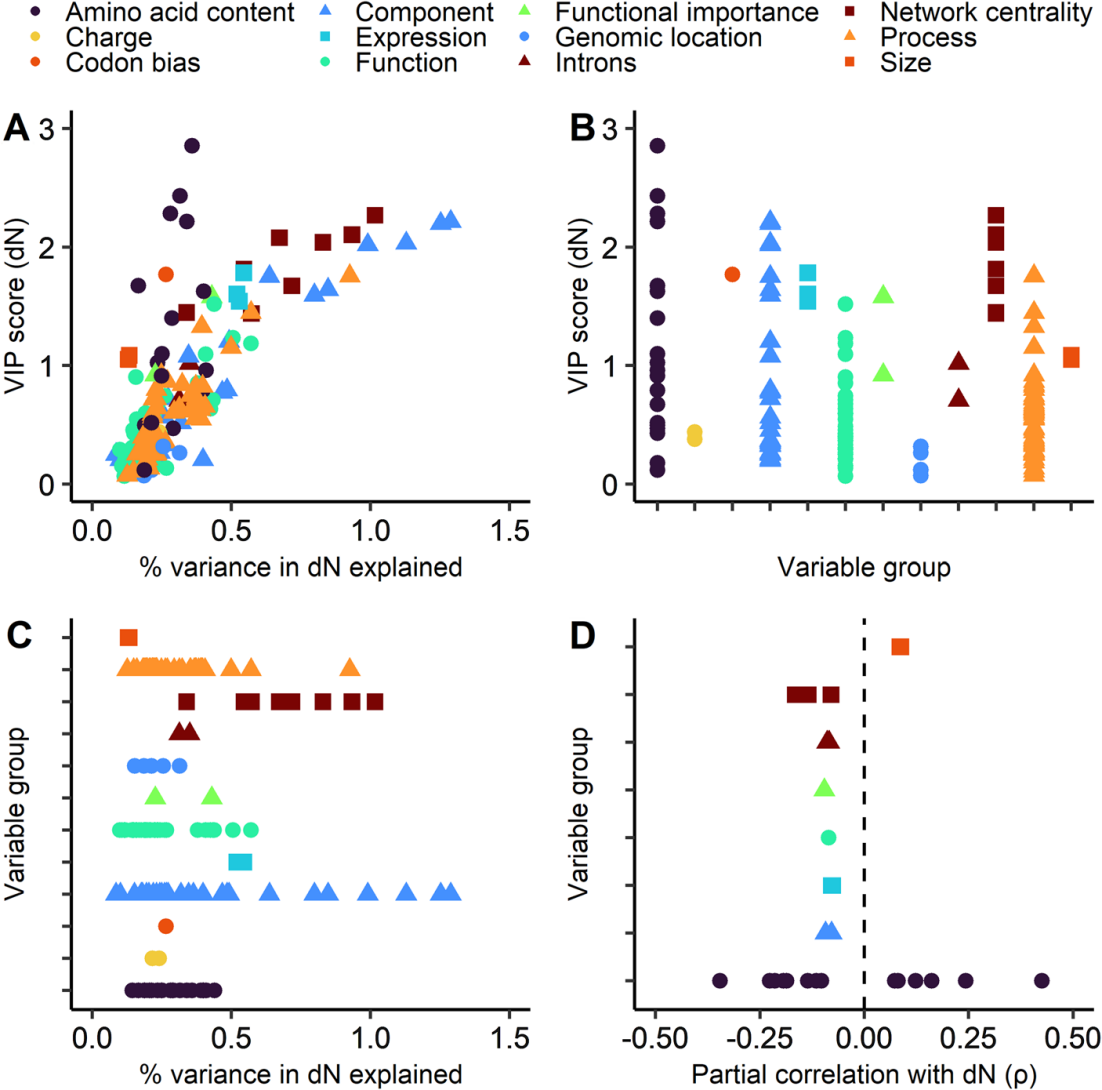
**A** Percent variance in d*N* explained by each variable using principal components regression compared to VIP scores for each variable in a partial least squares regression. Variables that are important in both models are closer to the top right corner. **B** VIP scores per variable in a PLS model with d*N* as dependent variable, grouped by variable group. **C** Percent variance explained per variable in a PCR model with d*N* as dependent variable, grouped per variable group. **D** Partial correlations (Spearman) between each variable and d*N*. Only significant correlations (Bonferroni-adjusted *p* < 0.05) shown

**Fig. 3:**
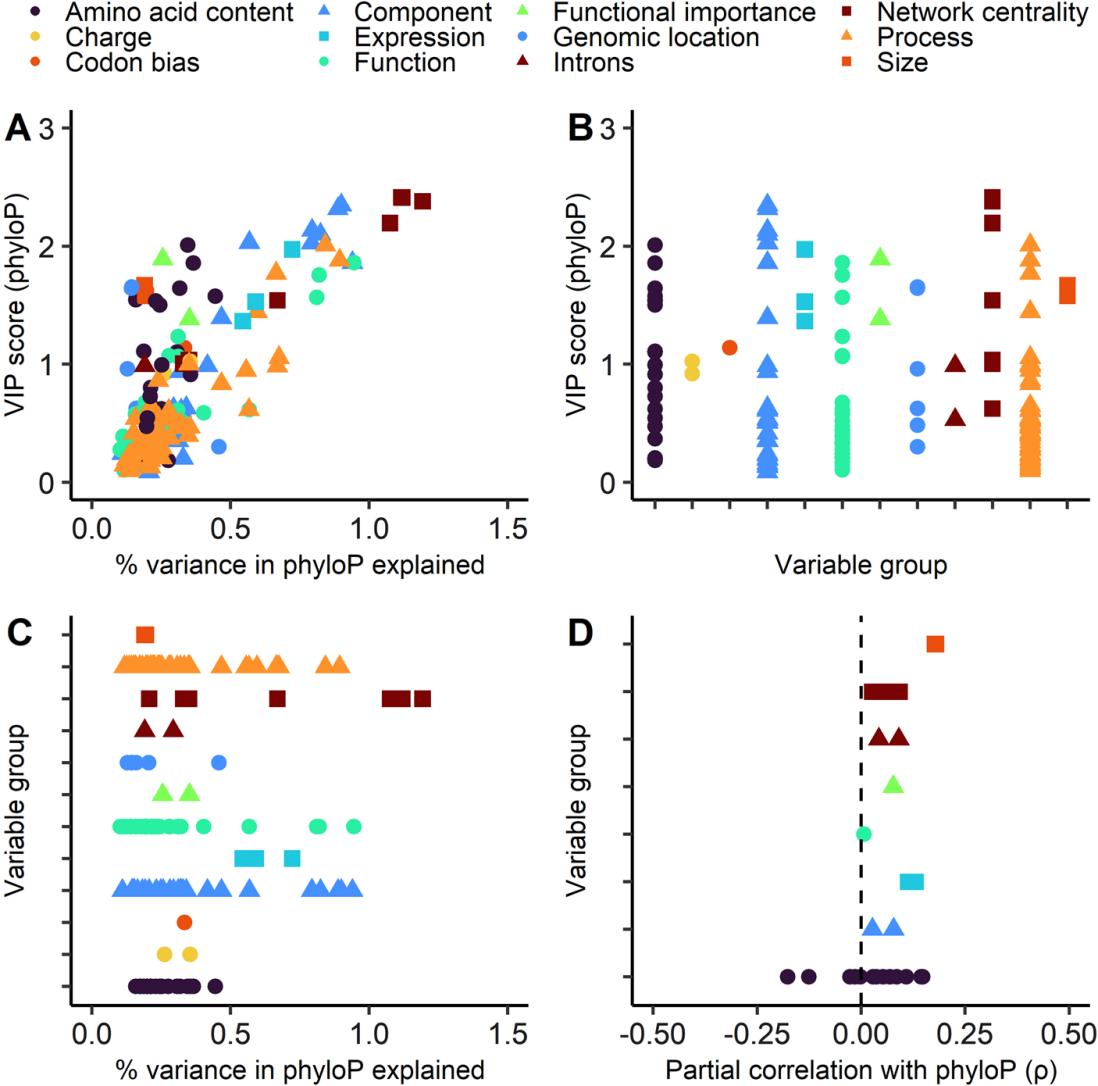
**A** Percent variance in phyloP explained by each variable using principal components regression compared to VIP scores for each variable in a partial least squares regression. Variables that are important in both models are closer to the top right corner. **B** VIP scores per variable in a PLS model with phyloP as dependent variable, grouped by variable group. **C** Percent variance explained per variable in a PCR model with phyloP as dependent variable, grouped per variable group. **D** Partial correlations (Spearman) between each variable and phyloP. Only significant correlations (Bonferroni-adjusted *p* < 0.05) shown

We used PLS and PCR models to assess gene-centric factors that affect gene conservation, using both d*N* and phyloP as metrics of sequence conservation. While these two metrics are correlated (ρ = − 0.524), they differ greatly in how they are calculated (see “Methods”). The independent variables included factors such as protein/mRNA gene expression values, protein features, gene features (size, introns, codon bias), and functional factors such as gene ontology assignment (cellular locations, processes, and functions); a full list of variables is available in the Figshare repository (https://doi.org/10.6084/m9.figshare.c.5263523.v6). Figures 2 and 3 illustrate that numerous variables each make a small contribution; no single variable explains more than 1.3% of the variance in either phyloP or d*N*. It would be possible to sum the VIP scores or percent variance explained per variable group; however, we believe that due to the number of individual variables this is likely to exacerbate model error and may artificially inflate the performance of variable groups with a higher number of individual variables.

As seen in both Figs. 2 and 3, network centrality is a very important variable determining sequence evolution rates. This is most apparent when considering phyloP, where four centrality metrics (functional degree centrality, physical degree centrality, functional eigencentrality, and physical eigencentrality) cluster in the top right corner in Fig. 3a, signifying that these variables were highly important in both the PLS and PCR models. With respect to d*N*, network centrality is also one of the most important variable groups in both regression models (Fig. 2a). All network centrality metrics except eigencentrality of physical interactions also show significant partial correlations (as strong as ρ = − 0.165, for functional degree centrality) with d*N* (Fig. 2d). For phyloP, correlations are weaker (no stronger than ρ = 0.092, also for functional degree centrality), but all correlations except the betweenness centrality metrics remain significant (Fig. 3d). Note that the opposite signs of correlations are expected since phyloP assigns higher values to more conserved genes, whereas for d*N* the opposite is true.

The fact that both eigencentrality (which measures the “importance” of the protein in the network) and degree centrality (which is a count of the number of interaction partners) are determined to be so important, and that the result is independent of whether only physical or all interactions are measured, indicates that network centrality constrains sequence evolution at multiple levels. Overall, it is apparent that network centrality is an important determinant of sequence conservation in *Schizosaccharomyces pombe* and that, in general, the more central the protein in the protein–protein interactions network, the more conserved its sequence is. We did not investigate this in other types of networks where the results are expected to be different, as discussed above.

### Amino Acid Composition Exerts a Significant Bidirectional Effect on Evolutionary Rates

According to the VIP scores calculated from the PLS model, amino acid composition is one of the most important variables affecting sequence evolution rates. The four variables with the highest VIP scores for dN are serine (VIP = 3.50), glycine (2.85), asparagine (2.43), and alanine (2.29) composition (Fig. 3b); by comparison the highest VIP score in the phyloP PLS model is 2.41 (for functional degree centrality). Amino acid composition is relatively less important in modelling phyloP—the highest VIP score for amino acid is 2.01 for alanine content—but is nonetheless still highly significant in that model. As seen in Figs. 2d and 3d, many amino acid composition variables correlate strongly and significantly either positively or negatively with constraint. It is interesting that the PCR models do not indicate that amino acid composition is a particularly important variable, and this highlights the benefits of using more than one modelling approach.

It is known that the changeabilities of amino acids differ depending on the structural requirements of the protein domain—particularly, there is a considerable difference in amino acids composition between transmembrane and other protein domains (Tourasse and Li 2000). It is also known that alanine and glycine, which both correlate relatively strongly positively with constraint (ρ = − 0.226 with dN and ρ = 0.144 with phyloP for alanine content), are enriched in highly conserved Low Complexity Regions (LCRs) (Ntountoumi et al. 2019). However, serine correlates strongly negatively with constraint (ρ = 0.425 with d*N* and ρ = 0.176 with phyloP) but is nonetheless also common in LCRs (Radó-Trilla and Albà 2012). Regardless, it is clear that amino acid composition serves as a proxy for protein domains and regions that are under different selective pressures.

### Intracellularity is a Very Important Determinant of Constraint

Intracellularity is the single variable that explains the most variance in d*N* (1.29%), which is supported by the high percent variance explained in phyloP (0.89%) as well as high VIP scores (2.21 for d*N* and 2.32 for phyloP). As seen in Figs. 2a and 3a, several cellular location (or “component”) variables cluster towards the top right corner; these are all specific intracellular locations such “cytoplasm” and “organelle”, which most likely reflects the strong effect of intracellularity on constraint. While only two cellular location variables significantly correlate with both d*N* and phyloP—cytosolic location (ρ =  − 0.093 with d*N*; ρ = 0.078 with phyloP) and cytoplasmic location (ρ =  − 0.079 with dN; ρ = 0.027 with phyloP)—both of these components reflect intracellularity. This shows that although covariates do appear to inflate the importance of cellular location in the regression models, there is clearly a direct effect of location. This is believed to be due to the complexity of the intracellular environment constraining evolution, extracellular communication changing more rapidly, and because extracellular proteins are pathogen targets necessitating positive selection (Julenius and Pedersen 2006).

Other gene ontology classifications also have a considerable impact. With respect to both d*N* (Fig. 2b, c) and phyloP (Fig. 3b, c), some process and function variables score comparably with some network centrality metrics. This largely reflects that proteins involved in key synthetic, transcriptional, and translational pathways are conserved between *Schizosaccharomyces* species—which is hardly unexpected. For instance, it is known that ribosomal components are some of the most conserved sequences across the tree of life (Isenbarger et al. 2008).

### Gene Expression, Length, and Functional Importance Each Have a Moderate Impact

As is clear in Figs. 2 and 3, many variables have some degree of influence, or have been influenced by, the degree of constraint. As expected, gene and protein expression levels were found to be highly significant variables in determining sequence conservation. However, this variable is clearly not the single most important in *S. pombe*. This is most apparent in Figs. 2a and 3a; several other variables are closer to the top right corner than expression variables are. Nonetheless, the expected correlations between d*N* and expression (ρ =  − 0.079) as well as between phyloP and expression (ρ = 0.131) are present and relatively strong.

Another variable that was expected to have considerable influence was sequence size (i.e., gene or protein length or mass)—size variables have a rather high VIP scores in the PLS models; gene length scores up to 1.66 in the phyloP PLS model, and up to 1.05% variance explained in the d*N* model. Indeed, gene length is the variable with the strongest positive partial correlation with phyloP (ρ = 0.180), although strangely gene length also correlates positively with d*N* (ρ = 0.086); these results directly contradict each other. As mentioned, previous studies on the topic have found both positive and negative correlations with ω, so we are not entirely surprised that two different metrics indicate opposite relationships. The existence of a correlation (positive or negative) between gene length and conservation is probably best explained by that the Hill–Robertson effect increases with gene length (Ingvarsson 2007), making selection on longer genes less effective. It remains unsolved why the direction of correlation varies.

As is well now established, functional importance is counterintuitively not the single most important determinant of constraint, but it is clearly not insignificant either. Essentiality correlates significantly with constraint measured as either d*N* (ρ =  − 0.096) or phyloP (ρ = 0.077), and while this variable explains a relatively moderate amount of variance (0.430% in d*N* and 0.255% in phyloP), it has high VIP scores (1.58 for d*N* and 1.89 for phyloP). This moderate influence is consistent with the current view of functional importance as a moderate determinant of constraint (Zhang and Yang 2015).

### Our Models Explain Over 50% of the Variance in d*N*

So far, the previous studies we are familiar with have only been able to explain less than 50% of variance in ω. Indeed, Drummond et al. (2006) are an outlier with 45% of variance in d*N*/d*S* explained in yeast; Alvarez-Ponce et al. (2019) only explain 22% of variance in yeast d*N*/d*S*, and an integrated analysis of multiple organisms only managed to explain 18% of variance (Alvarez-Ponce et al. 2017). Needless to say, these results are not directly comparable with ours, as we used phyloP and d*N* rather than ω, but it does illustrate the challenge of modelling the variables influencing evolutionary rates. Yang and Gaut (2011) modelled d*N* and d*S* in *Arabidopsis* separately, finding 11% and 21% of the variance in each explained, respectively. As seen in Table 1, our models explain over 30% of the variance in phyloP, and over 50% of the variance in d*N*.

**Table 1:** A comparison of the variance in dependent variable explained the holdout test set of three regression techniques applied to sequence evolution constraint prediction

	PLS (%)	PCR (%)	RF (%)
phyloP	32.4	31.0	42.4
d*N*	52.6	51.3	58.7

The RF model was trained to provide a comparison of model performance

*PLS* partial least squares, *PCR* principal components regression, *RF* random forest

Even as we have explained more than half of the variance in d*N*, and almost a third of the variance in phyloP, we think that it would be possible to improve these models further. We were unable to find any atlas of chaperone interactions in *S. pombe*, which Alvarez-Ponce et al. (2019) found to be very important as a determinant of constraint in *Saccharomyces cerevisiae*. We also do not investigate the role of network topology other than the protein–protein interactions network centrality, and we do not consider specific subnetworks where centralities are likely to have different effects. Adding these variables would also bring us closer to understanding how much the rate in sequence evolution depends on stochastics, which we believe is very likely to be the single most important factor that affects evolutionary rate.

## Conclusion

We show that the three most important known variables influencing rates of evolution (measured as d*N* or phyloP) in *Schizosaccharomyces pombe* are centrality in the protein–protein interactions network, amino acid composition, and cellular location; specifically, intracellularity, although these only explain a fraction of the variance in constraint. Many other variables have a weak to moderate influence. Our models explain about 1/3 of the variance in phyloP and half of the variance in d*N*, and including additional or more refined variables might reveal how much of the rate of evolution is determined by specific biological factors and how much of it is the result of stochastics.

## Data Availability

All data are provided both in their original format and processed on Figshare (https://doi.org/10.6084/m9.figshare.c.5263523.v6). The dataset from Grech et al. 2019 was retrieved from Figshare (https://figshare.com/articles/dataset/gene-based_data/6265748). Intron and genomic location data were from AnGeLi (http://bahlerweb.cs.ucl.ac.uk/AnGeLiDatabase.txt). Protein data were retrieved from PomBase (ftp://ftp.pombase.org/pombe/Protein_data/PeptideStats.tsv and ftp://ftp.pombase.org/pombe/Protein_data/aa_composition.tsv). Protein interactions data were retrieved from STRING v11 (https://stringdb-static.org/download/protein.links.v11.0/4896.protein.links.v11.0.txt.gz).

## Code Availability

All scripts are available both in the Figshare repository (https://doi.org/10.6084/m9.figshare.c.5263523.v6) and on GitHub (https://github.com/simonharnqvist/pombe).
